# The glocalization of antimicrobial stewardship

**DOI:** 10.1186/s12992-019-0498-2

**Published:** 2019-09-09

**Authors:** Olivier Rubin

**Affiliations:** Global Studies, Department of Social Sciences and Business, Roskilde University, Roskilde, Denmark

**Keywords:** Antimicrobial resistance, Governance, Glocalization

## Abstract

This brief commentary argues that *glocal governance* introduces a fruitful new perspective to the global governance debate of AMR, and cautions against too strict a focus on establishing globally binding governance regimes for curbing AMR.

## Background

Much attention has recently been devoted to finding ways to strengthen global governance mechanisms of AMR. The call for implementing binding global commitments and strengthening global institutions has figured prominently in this debate. Less attention has been paid to the tensions between global and local solutions. These tensions are particularly prominent in AMR stewardship, i.e., in the processes of formulating policies and strategies to deal with AMR. This commentary argues for an increased focus on glocal governance to strengthen AMR stewardship.

Calls for increased global antimicrobial stewardship to curb antimicrobial resistance (AMR) have been heeded in recent years. Many have pushed for legally binding international agreements and the establishment of a supranational entity dedicated to curbing AMR. The director of the WHO’s AMR Secretariat argued in a co-authored article in *Infectious Diseases* that legally binding governance mechanisms are necessary for strong, swift, and coordinated action to curb AMR [1]. A multi-stakeholder statement on AMR published in *Lancet* emphasized the need for binding legal agreements and establishing a novel supranational entity to facilitate such agreements [2]. Several prominent medical researchers have echoed the call for convening an intergovernmental panel on AMR [3]. Most recently, IACG’s report to the UN Secretary-General recommends adopting and implementing global standards and establishing an Independent Panel on Evidence for Action against Antimicrobial Resistance [4].

Interestingly, all these calls included comparisons to climate change. Indeed, the similarities between AMR and climate change are striking: both have adverse consequences today that might potentially turn catastrophic in the future; both risk tragedies of the commons where the benefits from antibiotics and emissions are local but the costs of resistance and climate change are global; both contain clear ethical dilemmas where some overuse a common resource (to the detriment of all of us) while others lack access to the resource; and responding to both threats includes high complexity and multiple constituencies.

However, one key lesson to take away from the international efforts to curtail climate change is that binding commitments are not necessarily effective. Several key states opted out of these climate treaties. Even the most prominent global governance effort, the 1997 Kyoto protocol, had only 37 industrialized countries committed to legally binding reductions in emissions, and they have mixed results to show for it [5]. Legally binding commitments were abandoned altogether in the recent Paris Agreement, in favor of self-imposed political promises. Other more successful international treaties have dealt with more restricted challenges that are cheaper to address, such as the Montreal Protocol to protect the ozone layer. Or they have dealt with multi-national health challenges rather than transnational ones, such as the Framework Convention on Tobacco Control (smoking in Canada does not have health implications in the US). Rather than broad calls for legal commitments and supranational bodies, a more pragmatic approach to AMR stewardship might be merited.

The global challenge of AMR, more so than with climate change, requires solutions that are compatible with local needs and contexts. The equivalent to universal emissions targets is hard to find in the realm of AMR. In some countries, the most effective solution may be to provide incentives for patients, doctors, and farmers to reduce their use of antibiotics. In other countries, access to clean drinking water or fast diagnostics matter more than adjustments in antibiotic consumers’ behavior. This marked variability in both the state of public health and type of health challenge necessitates a broader pallet of solutions than the binary climate change option of either restricting the consumption of energy or lowering emission intensity. Hence, a combination of non-binding and binding agreements at multiple levels and across different sectors are more likely to be effective than the more comprehensive, wide-scoped treaties that we know from climate change. The analytical lens of *glocalization* can provide insights on how to establish such more complex global governance regime.

Examining AMR stewardship through the prism of glocalization places an emphasis on tensions between the universal (global) and the particular (local) [6]. Glocalization acknowledges that global processes are not happening against or outside local forces; on the contrary, both global and local are mutually constituent concepts. *Glocal governance*, therefore, suggests that global challenges should be addressed by the effective reconciliation of the local and the global. Three concrete strategies can help facilitate a strong glocal governance regime of AMR.

First, from the perspective of glocal governance, effective AMR stewardship might be achieved by policy diffusion where ‘frontrunner’ countries or institutions set best practices/ideas that spread across nations and are adapted to local contexts [7]. While agenda-setting at the global level has been shown to stimulate local policy innovation and adaptation, top-down control regulation based on universal standards risks curbing this organic policy diffusion between nations and institutions. The international push for National Action Plans (NAPs) on AMR provides a welcome opportunity for policy diffusion. More than 115 countries have now developed these plans by drawing global guidelines and recommendations. Yet, NAPs also allow countries to balance and integrate AMR stewardship with other country-specific priorities such as economic development, universal health care, unemployment, food security, etc. This increases the prospects for political support in low and middle income countries where access to antibiotics is often a more severe health concern than AMR. Currently, however, the actual implementation of these NAPs is sluggish. Less than 20% of NAPs are under implementation with funding and monitoring mechanisms in place. The diffusion of policies, ideas and practices matters less in situations where governments lack the capacity and political attention to put them to practice. Strong glocal governance of AMR, therefore, needs to extend beyond leveraging policies and practices through NAPs.

Second, an effective glocal governance strategy of AMR might rely on *polycentric governance* in the form of horizontal collaboration on a subnational level [8]. A global governance unit within a hierarchical structure will often be poorly situated to identify, assess and solve issues that are heavily influenced by national, regional, and local dynamics. Instead, polycentric governance builds on a network of multiple and transboundary governing authorities capable of drawing on local knowledge, mutual monitoring, and peer learning. Policy sustainability and effectiveness is ensured by the fact that the initiative and implementing capacity originate from the same bottom-up network of subnational stakeholders. Such a polycentric network allows for a greater sense of shared responsibility of goals across countries, sectors and actors. These networks could work independently of global and national action plans. Inspired by the success of the C40 network that connects 94 megacities committed to addressing climate change, for example, a global network could link major national health care facilities that are committed to addressing AMR. An obvious venue would be forming stronger horizontal ties between the many national AMR centers that have recently been established around the globe.

Lastly, *Problem-Driven Iterative Adaptation* (PDIA) is a glocalization strategy that focuses on solving specific problems in particular local contexts rather than transplanting “best practice” solutions [9]. It relies on a gradual approach to solving problems that encourages experimentation; active learning mechanisms with tight feedback loops; and the inclusion of broad sets of actors to ensure that reforms are viable, legitimate and relevant. The gradual problem-driven strategy is somewhat akin to a salami tactic in that policies should aim to address small-scale country-specific problems related to AMR. These problem-driven policies can benefit from greater specificity with regards design, implementation, monitoring and surveillance than is possible in universal treaties and conventions. Even at the local level, tackling AMR requires sustained and coordinated action across a range of institutions and sectors, including human and animal health, food production, environment, water and sanitation, education and trade. Integrating health, agricultural and industrial actors in ways that encourage innovation and learning is essential for effective and viable AMR policies. Sweeping global agreements could risk crowding-out the alternative ideas and initiatives that emerge when these local agents engage in problem solving. Narrower-scoped legal agreements such as tailored restrictions on the use of antibiotics in healthy livestock or limits on antibiotic residue in wastewater are more easily integrated into PDIA.

The WHO has produced several useful scenarios for stronger global governance of AMR [10]. Glocal antimicrobial stewardship is likely to complement these global actions by offering a broader topography of governance options that directly address the many and varied local health challenges contributing to AMR. The commentary highlighted three concrete strategies, namely policy diffusion, polycentric governance and PDIA, to broaden the perspective of how to facilitate effective governance regimes (in plural) of AMR. Pursing glocal governance solutions may spur countries to act and provide useful guidance for national and local initiatives of AMR without imposing too many expensive and ultimately ineffectual international obligations.

## Conclusions

The global challenge of AMR requires solutions that are compatible with local needs and contexts. Glocal governance might constitute an effective international regime of antimicrobial stewardship. Three concrete strategies can help facilitate a strong glocal governance regime of AMR: (i) policy diffusion where agenda-setting at the global level stimulates (but does not dictate) local policy innovation and practices; (ii) polycentric governance where subnational agencies form horizontal networks to solve particular problems; and (iii) problem-driven iterative adaptation practices that activate multiple levels of governance and sectors in addressing country-specific problems related to AMR.

## Data Availability

n/a
